# Autoimmune Polyglandular Syndrome Type II: A Case Report

**DOI:** 10.7759/cureus.31641

**Published:** 2022-11-18

**Authors:** Hera Jamal, Michael LaLoggia, Neil Harjai

**Affiliations:** 1 College of Osteopathic Medicine, Lake Erie College of Osteopathic Medicine (Bradenton Campus), Bradenton, USA; 2 College of Osteopathic Medicine, Lake Erie College of Osteopathic Medicine, Erie, USA; 3 Psychiatry, Rochester Regional Health, Rochester, USA

**Keywords:** primary adrenal insufficiency, hypothyroidism, adrenal crisis, autoimmune endocrine dysfunction, polyendocrinopathy, polyendocrinopathies, autoimmune polyglandular syndrome type ii, addison's disease, schmidt syndrome, autoimmune polyglandular syndrome

## Abstract

Autoimmune polyglandular syndromes (APS) are polyendocrinopathies characterized by autoimmune dysfunction of multiple endocrine organs. We present the case of a 23-year-old male with a past medical history of autoimmune thyroiditis diagnosed seven months prior who presented with a chief complaint of six months of fatigue, shortness of breath, and weight loss. Physical exam was remarkable for global hyperpigmentation, notably of his palmar creases and gingiva. The patient was also tachycardic and hypotensive. He initially received two liters of 0.9% NaCl and 10 mg of intravenous dexamethasone. Random cortisol was <0.5 g. A cosyntropin test showed an insufficient increase in cortisol in response to adrenocorticotropin hormone (ACTH), confirming the diagnosis of primary adrenal insufficiency (AI). A computed tomography (CT) scan of the abdomen was negative for adrenal hemorrhage. A sexually transmitted disease (STD) panel was obtained to rule out the infectious cause and was negative. The patient was given glucocorticoids and his symptoms improved with fluid and electrolyte supplementation. This case report highlights the importance of close monitoring of patients with autoimmune endocrine abnormalities. These patients should be followed by an endocrinologist every six months for prompt diagnosis and risk mitigation.

## Introduction

Autoimmune polyglandular syndromes (APS) are rare polyendocrinopathies that are characterized by autoimmune dysfunction of multiple endocrine organs. Non-endocrine manifestations are not required for diagnosis but have been described in the context of these syndromes. 

APS-II involves primary adrenal insufficiency (AI), often referred to as Addison’s disease, concomitant with autoimmune thyroiditis and/or type I Diabetes Mellitus (T1DM) [1,2]. It was first characterized in 1926 by Schmidt, who noted two patients with autoimmune adrenal insufficiency and chronic lymphocytic thyroiditis (Schmidt’s syndrome) [3]. It should be noted that either autoimmune hypothyroid conditions (i.e., Hashimoto’s thyroiditis), or hyperthyroid conditions (i.e., Grave’s disease), may be described in APS-II [2]. There is no single mutation that is accountable for APS-II, and it is considered to have a multifactorial polygenic inheritance. We present a case of autoimmune polyglandular syndrome type II, which illustrates the need for clinicians to assess for multiple autoimmune endocrinopathies once one is diagnosed. 

## Case presentation

A 23-year-old male with a past medical history of autoimmune thyroiditis arrived at the emergency department with a chief complaint of six months of fatigue, shortness of breath, weight loss, and hyperpigmentation. He also endorsed one to two weeks of diarrhea, abdominal pain, chills, myalgias, and salt cravings. He denied any fever, vision change, voice change, chest pain, palpitations, or joint swelling. Seven months prior, he had presented with lethargy, myalgias, and shortness of breath. He was found to have a thyroid stimulating hormone (TSH) level of 15.95 (Ref 0.35-5.50 uIU/mL) and an anti-thyroid peroxidase antibody level >1300 (Ref: <60 IU/mL) and was subsequently diagnosed with Hashimoto’s thyroiditis. At that time, he was prescribed 50 mcg of levothyroxine daily, which improved his hypothyroid symptoms. Upon admission, vitals were significant for tachycardia of 110 beats per minute and blood pressure of 87/55. In the emergency department, the patient received two liters of 0.9% NaCl along with 10 mg of intravenous dexamethasone. Labs drawn in the emergency department were significant for leukopenia, mild hyponatremia, elevated creatinine, and mild transaminitis (Table 1).

**Table 1:** Laboratory values in the emergency department

Lab	Value	Reference Value
White blood cells	3.6	4.0-10.0 x 10^3/µL
Sodium	134	136-146 mEq/L
Potassium	4.6	3.5-5.1 mEq/L
Creatinine	1.4	0.6-1.2 mg/dL
ALT	51	10-49 U/L
AST	52	7-37 U/L
Adrenocorticotropic hormone (ACTH)	<5 pg/mL	9-52 pg/mL (AM)
AM cortisol	<0.5 mcg	5.0-23.0 mcg/dL

Physical exam was remarkable for evenly hyperpigmented skin, with notable hyperpigmentation in the creases of his palms and on his gingiva. 

Clinical course

**Figure 1:**
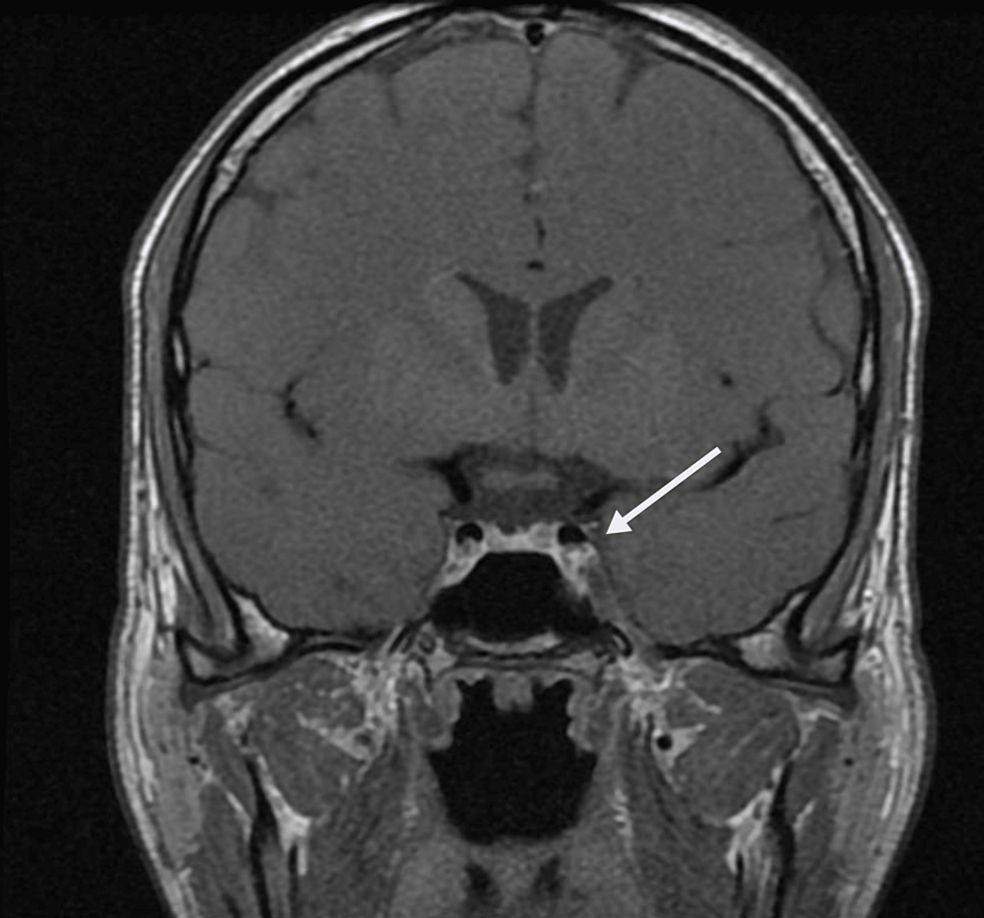
Magnetic resonance imaging (MRI) of head obtained during hospital course

A cosyntropin test showed an insufficient increase in cortisol in response to adrenocorticotropic hormone (ACTH) after 30 and 60 minutes. Cortisol level before the cosyntropin test was <0.5 ug (Ref: 3.0-23.0 ug/dL) and rose to only 6.2 ug/dL (Ref: >7 ug/dL) after 30 minutes and 10.2 ug/dL (Ref: >18 ug/dL) after 60 minutes. A computed tomography (CT) scan of the abdomen was negative for any source of adrenal hemorrhage. Magnetic resonance imaging (MRI) of his head showed a rounded 4 x 5 mm hypo-enhancing focus in the left pituitary gland, likely representing a pituitary microadenoma (Figure 1). There was no evidence of infarction, intracranial hemorrhage, mass effect, or hydrocephalus. A sexually transmitted disease (STD) panel was obtained to rule out an infectious cause of adrenal insufficiency and was negative. His adrenal insufficiency was addressed via the initiation of five mg of prednisone daily as an inpatient, and his symptoms continued to improve with fluid and electrolyte supplementation. He was discharged after two days with the knowledge that he would need daily hydrocortisone and was counseled on the importance of wearing an Addison’s alert band.

## Discussion

APS-II is a well-characterized polyglandular endocrine disorder with an estimated prevalence of 1:1,000-1:20,000 [3,4]. The average age of onset is 20-40, and it is thrice more likely to occur in women than men [3]. APS-II has multifactorial inheritance and has a known association with human leukocyte antigens (HLA) DR3, DR4, DQ2, and DQ8 [3]. 

**Figure 2:**
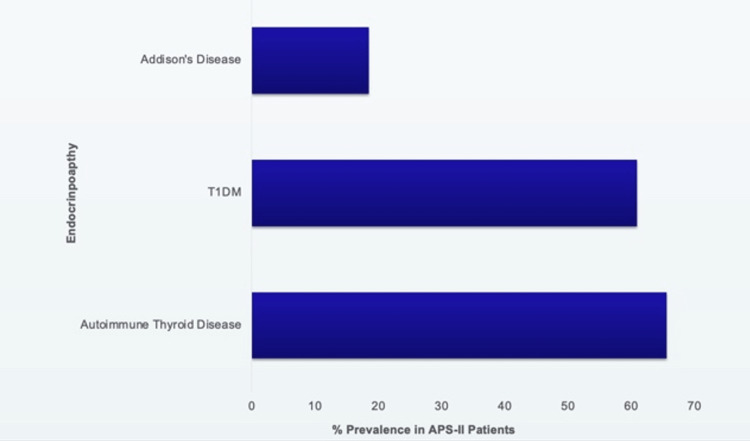
Prevalence of autoimmune endocrinopathies in long term study of 151 Individuals with APS-II Original graphic displaying the prevalence of endocrine manifestations seen in autoimmune polyglandular syndrome type II (APS-II). These include Addison's Disease, Type 1 Diabetes Mellitus (T1DM), and autoimmune thyroid disease. Created with information from a long-term study of individuals with APS-II [5].

**Figure 3:**
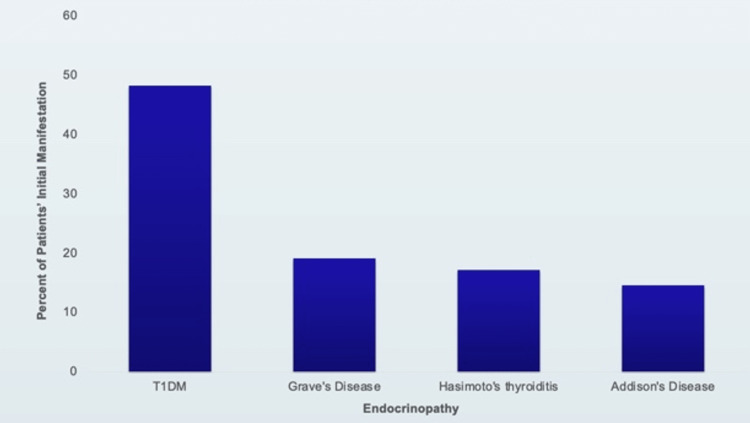
First disease manifestation in patients with APS-II in the long-term study of 151 individuals with APS-II Original graphic displaying initial manifestations of autoimmune polyglandular syndrome type II (APS-II), including Addison's Disease, Type 1 Diabetes Mellitus (T1DM), Hashimoto's thyroiditis and Grave's disease. Created with information from a long-term study of individuals with APS-II [5].

From a cohort of 151 patients with APS-II [5], the prevalence of each disease and the first endocrine manifestations are displayed in Figures 2, 3, respectively. Autoimmune thyroid disease was seen more often than any other endocrine disorder in 66% of patients. Type 1 diabetes was the first manifestation of APS-II in just under half (48%) of these patients. In addition, autoimmune thyroid disease was seen in combination with diabetes more often than any other two abnormalities [5,6]. 

The time interval between manifestations of different endocrinopathies in APS-II has been shown to have considerable variability. The longest time interval (13.3 ± 11.8 years) was noted between type 1 diabetes and thyroid disease [5], respectively. A much shorter time was observed between Addison’s disease and thyroid disease [5], respectively. It was also noted that when thyroid disease was present as the first component of the syndrome, there was a relatively short period before the development of a second endocrinopathy [5]. 

**Figure 4:**
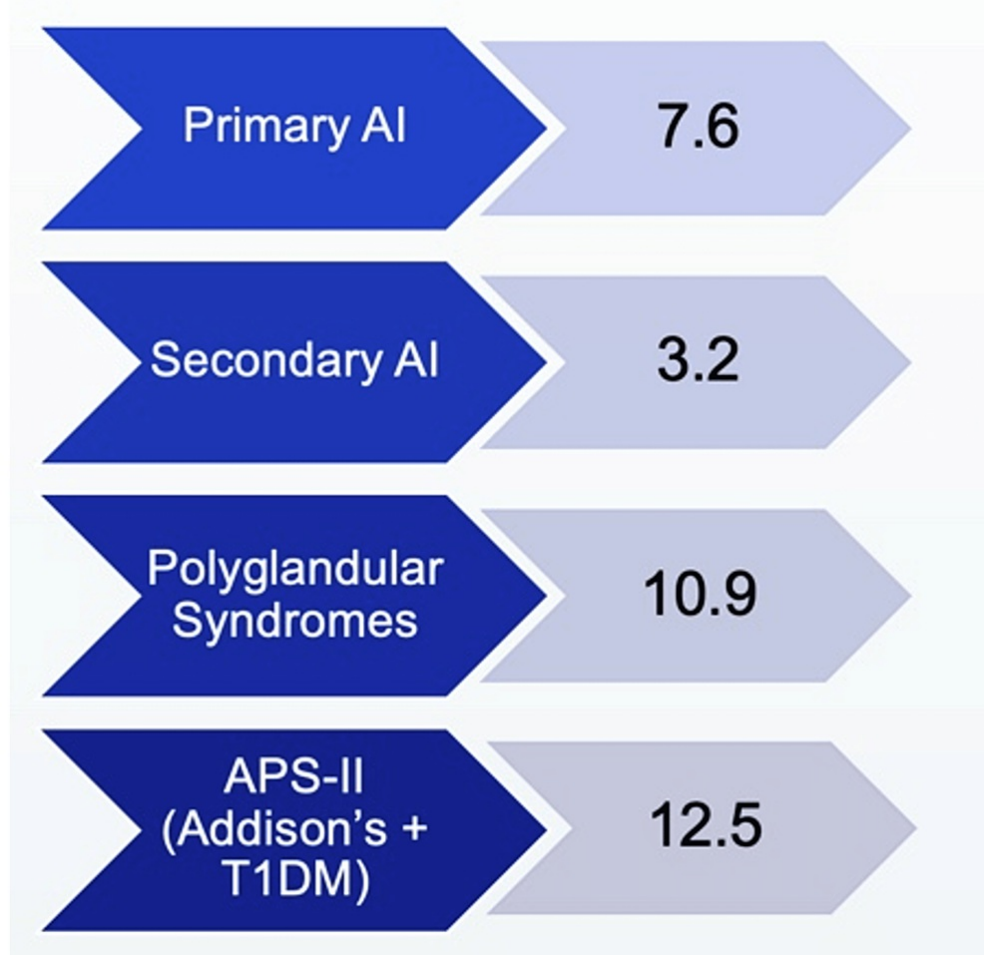
Prevalence of adrenal crises in patients with different etiologies of adrenal insufficiency. Original graphic displaying prevalence of adrenal crises per 100 patient years in patients with different etiologies of adrenal insufficiency (AI), including primary AI, secondary AI, polyglandular syndromes, and autoimmune polyglandular syndrome type II (APS-II). Analysis of data obtained from large German healthcare provider [6].

Of patients diagnosed with Addison’s disease, 50% might get diagnosed with another autoimmune condition in their lifetime [7]. Of note, people with APS-II are over two and a half times more likely to have an adrenal crisis when compared to those only diagnosed with Addison’s disease or secondary adrenal insufficiency alone [8] (Figure 4). For example, in a patient with known autoimmune hypothyroidism, 1% will go on to develop adrenal insufficiency [1]. Thus, a diagnosis of APS-II should confer a higher degree of suspicion for adrenal crisis and may ensure more prompt life-saving treatment. 

## Conclusions

This case report highlights the importance of close monitoring of patients with one autoimmune disorder of endocrine origin. The diagnosis of APS-II is often delayed due to existing comorbidities and non-specific symptoms at presentation. A lower threshold of suspicion should be applied to patients with a pre-existing autoimmune disease when considering the possibility of another autoimmune condition.

There is sufficient evidence to suggest that secondary endocrinopathies can present relatively quickly in patients with already-established autoimmune endocrine disorders. Additionally, once a patient is diagnosed with APS-II, they have a significantly higher likelihood of developing an adrenal crisis. Therefore, these patients must be followed-up regularly for prompt diagnosis and risk mitigation.
